# Identification of Key Gene and Pathways for the Prediction of Peritoneal Metastasis of Gastric Cancer by Co-expression Analysis

**DOI:** 10.7150/jca.39645

**Published:** 2020-03-04

**Authors:** Simeng Zhang, Dan Zang, Yu Cheng, Zhi Li, Bowen Yang, Tianshu Guo, Yunpeng Liu, Xiujuan Qu, Xiaofang Che

**Affiliations:** 1 Department of Medical Oncology, the First Hospital of China Medical University, Shenyang 110001, China;; 2 Key Laboratory of Anticancer Drugs and Biotherapy of Liaoning Province, the First Hospital of China Medical University, Shenyang 110001, China;; 3 Liaoning Province Clinical Research Center for Cancer, Shenyang 110001, China

**Keywords:** gastric cancer, peritoneal metastasis, WGCNA, MSRB3, PI3K-Akt

## Abstract

Peritoneal metastasis is the most common pattern in advanced gastric cancer and can predict poor disease prognosis. Early detection of peritoneal tumor dissemination is restricted by small peritoneal deposits. Therefore, it is critical to identify a novel predictive marker and to explore the potential mechanism associated with this process. In the present study, one module that correlated with peritoneal metastasis was identified. Enrichment analysis indicated that the Focal adhesion and the PI3K-Akt signaling pathway were the most significant pathways. Following network and Molecular Complex Detection (MCODE) analysis, the hub-gene cluster that consisted of 19 genes was selected. Methionine sulfoxide reductase B3 (*MSRB3*) was identified as a seed gene. Survival analysis indicated that high expression levels of *MSRB3* were independent predictors of peritoneal disease-free survival (pDFS) as determined by univariate (HR 8.559, 95% CI; 3.339-21.937; P<.001) and multivariate Cox analysis (HR 3.982, 95% CI; 1.509-10.509; P=.005). Furthermore, patients with high levels of *MSRB3* exhibited a significantly lower Overall Survival (OS) (log-rank P = 0.007). The external validation was performed by the (The Cancer Genome Atlas (TCGA)) (log-rank P = 0.037) and Kaplan Meier-plotter (KMplotter) (log-rank P = 0.031) data. *In vitro* experiments confirmed that MSRB3 was a critical protein in regulating gastric cancer cell proliferation and migration. In conclusion, High expression levels of *MSRB3* in GC can predict peritoneal metastasis and recurrence as well as poor prognosis. Furthermore, *MSRB3* was involved in the regulation of the proliferation and migration of GC cells.

## Introduction

Gastric cancer (GC) is the fifth most commonly diagnosed cancer and third leading cause of death worldwide. It is estimated that over 1,000,000 new cases and 783,000 deaths will be attributed to GC in 2018 according to demographic data 1. The incidence rates have been increased considerably in Eastern Asia. Peritoneal dissemination is a pattern of invasiveness and metastasis and is present in over 50% of patients with advanced gastric cancer despite radical surgery. This process can predict poor prognosis 2. Peritoneal involvement is considered a portent of disease recurrence 3. The treatment strategy of peritoneal metastasis includes chemotherapy, intraperitoneal perfusion and surgery, which exhibit limited efficacy. In addition, early diagnosis of peritoneal metastasis is also restricted by small peritoneal deposits, which are difficult to detect by imaging tests and biopsy 4. Therefore, identification of effective prediction markers of peritoneal metastasis is a vital part of improving survival outcomes in advanced gastric cancer.

Weighted Gene Co-expression Network Analysis (WGCNA) is an effective method of detecting co-expression modules and genes. It is a comprehensive collection of R functions for performing weighted correlation network analysis including functions for network construction, module detection, gene selection and calculation of topological properties. This approach has been frequently used in numerous aspects of cancer biology in order to identify candidate biomarkers or therapeutic targets 5-7. It can aid the comparison of differentially expressed genes and is also able to identify interactions among genes in different co-expression modules 8. The genes included in a module can be summarized with the module eigengene, which is considered a representative of the gene expression profile. The correlation between clinical parameters and module eigengenes is estimated to identify the modules related to these clinical variables.

The present study aimed to identify novel predictive markers and explore the potential mechanisms of peritoneal metastasis in gastric cancer. A total of 295 samples were analyzed by co-expression analysis based on WGCNA. The topological features of the protein-protein network analysis were constructed using the differentially expressed genes in the modules. The identification of the hub genes was also performed. This process may provide novel insight in the early detection and treatment targets of advance gastric cancer with peritoneal metastasis.

## Material and Methods

### Data collection and screening

Microarray data of GSE62254 were downloaded from the Gene Expression Omnibus (www.ncbi.nlm.nih.gov/geo/). Totally 300 gastric cancer sample data analyzed by HGU133plus2 Affymetrix chip was obtained. 295 samples with both gene expression and clinical pathological parameters were included. RNASeqV2 expression data and clinical data which download from The Cancer Genome Atlas (TCGA) portal (http://cancergenome.nih.gov/) and Kaplan Meier-plotter (KMplotter) (http://www.kmplot.com/) are designed as external validation of GEO datasets.

### Construction of co-expression network

Co-expression network was constructed by the WGCNA algorithm package in R project 7. Average connectivity and scale independence analysis of modules with different power value were performed by gradient test (power value ranging from 1 to 20). Appropriate power value was determined when the scale independence value was equal to 0.9. WGCNA algorithm was then used to construct the co-expression modules and extract the gene information in each module. For module detection, hierarchical clustering was used to produce a hierarchical clustering tree (dendrogram) of genes by function hclust based on dissTOM. The Dynamic Tree Cut method was performed for branch cutting to generate modules and a relatively large minimum module size of minClusterSize = 30. Modules with similar expression profiles were merged when the correlation of module epigengenes was greater than 0.75.

### Identifying modules associated with clinical traits and functional enrichment analysis

The correlation between module epigengens and clinical parameters including peritoneal seeding and ascites clinically significant was evaluated by Pearson's correlation tests, and p < 0.05 was considered to be significantly correlated. To investigate the potential biological themes and pathways of genes in the selecting modules, the clusterprofiler package 9 was used to annotate and visualize gene ontology (GO) terms and Kyoto Encyclopedia of Genes and Genomes (KEGG) pathways.

### Protein-Protein Interaction Network Construction and Analysis

Protein-protein interaction network (PPI) of selecting module was visualized by Cytoscape software (v3.6.1)^10^. Topological features of the network were calculated to screen the hub network. In order to identify the critical gene in the network, the modules in the PPI network were analyzed by MCODE (Molecular Complex Detection) plug-in in Cytoscape software with the default parameters “Degreed Cutoff = 2,” “Node Score Cutoff = 0.2,” “K-Core = 2” and “Max.Depth = 100” 11.

### Survival analysis

Statistical analyses were performed by using SPSS software 24.0. Survival curves were calculated by the Kaplan-Meier (KM) method and compared with the log-rank test. Univariate and multivariate analyses are analyzed by Cox proportional hazards regression models. Forward stepwise regression is performed to select the significant variables constitute to the final multivariable model. The prediction accuracy of Methionine sulfoxide reductase B3 (MSRB3) assessed by the time-dependent receiver-operating characteristics (ROC) curves and AUC curve. All statistic test in the present study were set as P<0.05 as statistical significance.

### Experimental validation

**Cell culture:** MKN45 gastric cancer cell lines was obtained from the Cell Bank of the Chinese Academy of Sciences (China) and grown in RPMI-1640 (GibcoBRL, USA) supplemented with 10% fetal bovine serum (FBS), penicillin (100 U/mL) and streptomycin (100 mg/ mL), in a humid atmosphere containing 5% CO 2 at 37°C.

**Reagents and Antibodies:** Anti-MSRB3 antibodies were obtained from Novus Biologicals (USA). Antibodies targeting Akt, phosphorAkt (Ser473), PI3-kinase were obtained from Cell Signaling Technology (USA). All the other antibodies were purchased from Santa Cruz Biotechnology (USA).

**Small Interfering RNA (siRNA) Transfections:** The MSRB3 siRNA sequences from Beijing GeneX Health technology Co., Ltd. (Beijing, China), were used: 5'-GACCGAAAGUGCCUUUGAATT-3'. The control sequence was UUCUCCGAACGUGUCACGU. The siRNAs were transfected with Lipofectamine 2000 (Invitrogen, Carlsbad, CA) per the manufacturer's instructions.

**MTT assay and Western blotting:** MTT assay and Western blot and immunoprecipitation were performed as described previously 12.

**Transwell migration assay:** The migration assay was performed by 24-well chemotaxis chambers (Corning, Corning, NY). The upper and lower cultures were separated by 8-μm pore sized polycarbonate filters. Gastric cancer cells transfected with MSRB3 siRNA or control siRNA were seeded at 1.25 × 10^5^cells/ml in serum-free 1640, and 200μl cell suspension was added to the upper chamber. Then 0.5 ml 1640 containing 2.5% FBS was added to the lower chamber. After incubation for 24 hours, the migrated cells adherent to the filters were fixed with ethanol and stained with Giemsa solution. The migrated cells were counted under bright-field microscope.

**Statistical analysis:** Data are reported as means ± SD. Student's t-test or one-way ANOVA were used to evaluate differences between or among groups. P < 0.05 was considered statistically significant. Each experiment was repeated at least three times.

## Results

### Patients characteristics and data processing

Gastric cancer data were derived from GSE62254. A sample size of 295 GC patients with their clinicopathological parameters and survival data were included in the present analysis (Table 1). The patient characteristics in GSE62254 were similar with those reported in previous randomized clinical trials 13, 14. The median age at diagnosis was 63 years old. The patients were classified according to the TNM status as follows: T2 (62.4%), N1 (43.4%) and M0 stage (90.8%). The TNM stage I, II, III, IV cases corresponded to 10.2%, 31.9%, 32.2% and 25.8% of the cohort, respectively. The documented recurrence rate was 42.0% and the most frequent recurrence site was the peritoneum (18.3%). A total of 5,118 genes were selected from 20,472 original probes for further analysis.

### Construction of co‑expression module of gastric cancer with peritoneal metastasis

**Figure 1:**
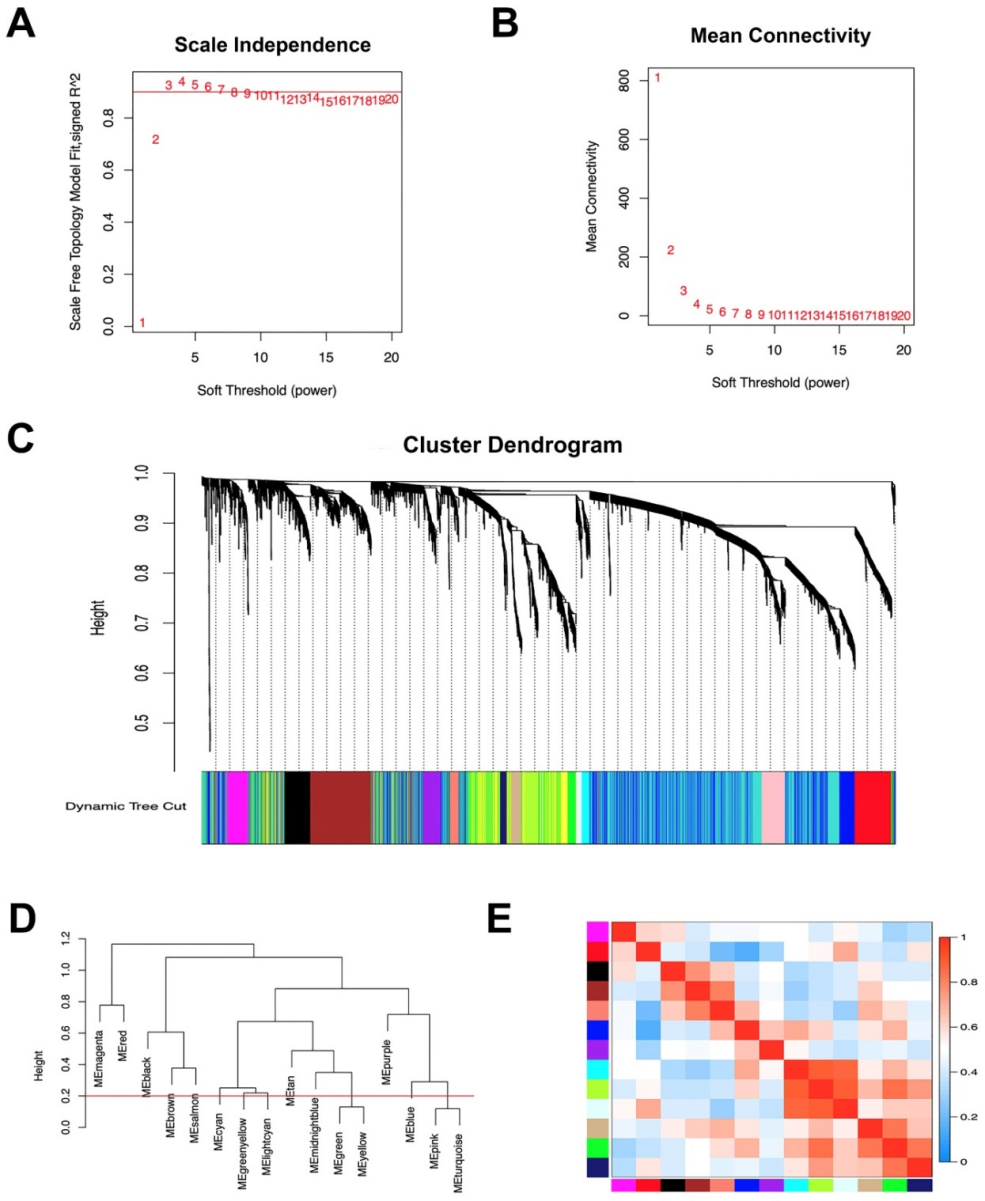
** Construction of co‑expression module of gastric cancer with peritoneal metastasis.** (A) The correlation of different soft threshold power values and scale independence of co-expression network. (B) The effect of soft threshold power values on mean connectivity of co-expression network. (C) The hierarchical cluster dendrogram was used to identify co-expression gene modules and each module was assigned with different colors. (D) Gene modules with similar expression profiles were merged according to the threshold (Red line) by calculating eigengenes of each module. (E) Heatmap plot of the adjacencies of modules. Red means positive correlation and blue means negative correlation.

The co-expression network was constructed using WGCNA. The power value is a vital parameter that can affect the independence and average connectivity degree of the co-expression modules. The network approximate scale-free topology distribution following selection of the appropriate soft threshold power equals 3 (Figure 1A, B). Therefore, the power value 3 was selected for further analysis. As a result, a total of 16 modules marked with different colors were identified by the Dynamic Tree Cutting method with a sensitivity deepSplit 2 in order to branch splitting (Figure 1C). Cluster analysis was performed by calculating the eigengenes of each module. The analysis aimed to identify modules with higher adjacency degree. Among 16 modules, there were three pairs of gene modules that were merged according to the threshold 0.2 (Figure 1D, E).

### Module-trait correlations and functional enrichment analysis

**Figure 2:**
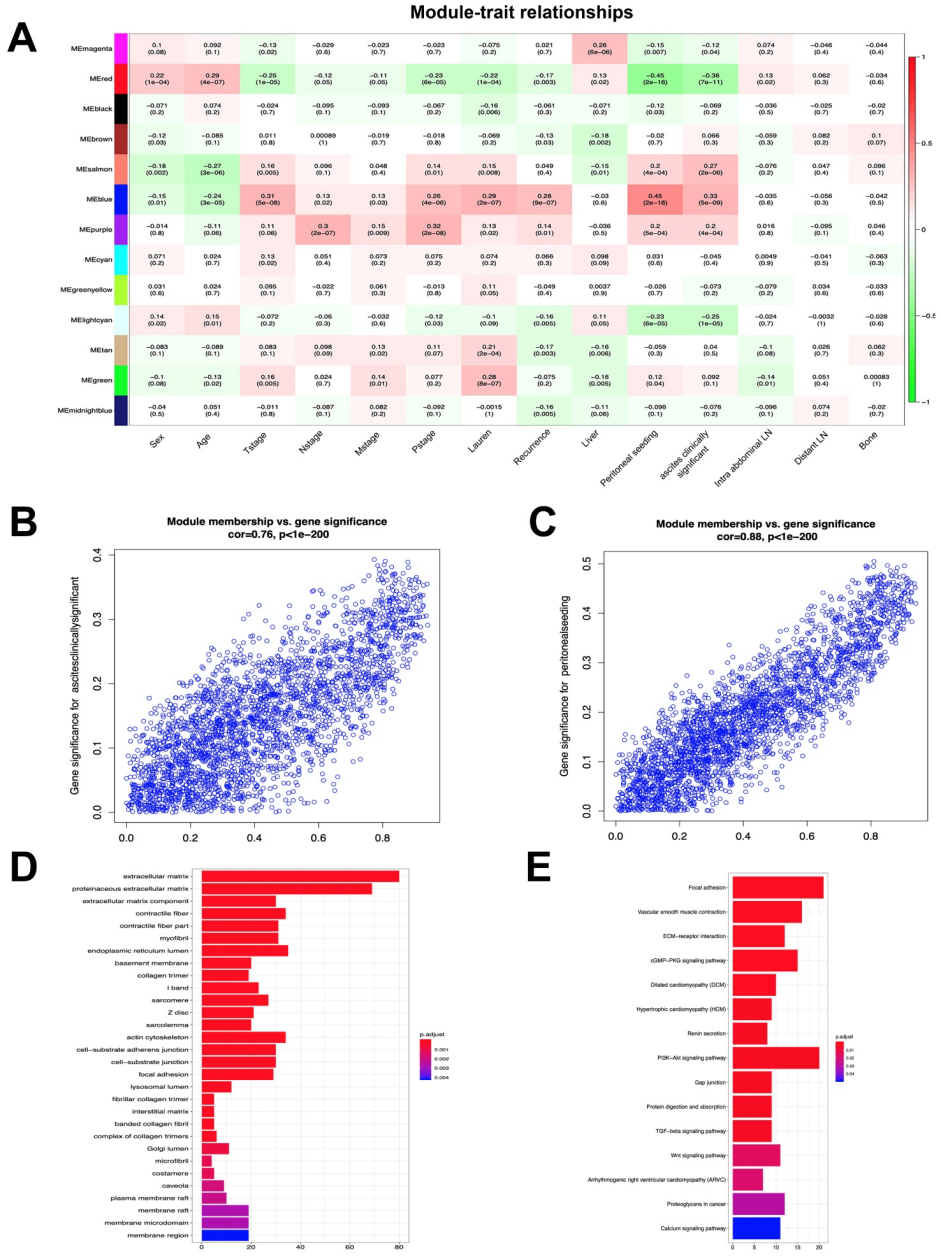
** Module-trait correlations and functional enrichment analysis.** (A) Each row corresponds to module eigengene, column to a clinical trait. Numbers in each table describe the correlation and p value of module eigengenes and trait. The table is color-coded by correlation according to the color legend. (B) The correlation of genes in blue module with trait of ascites clinically significant. (C) The correlation of genes in blue module with trait of peritoneal seeding. (D) Significantly enriched GO annotations of blue module. (E) Significantly enriched KEGG pathways of blue module.

To investigate the clinical significance of the module, we analyzed the correlation between clinical parameters and module eigengene. The results indicated that the blue, salmon, purple and green modules were positively associated with both peritoneal seeding and ascites. These parameters were used to define the peritoneal metastasis (Figure 2A). In addition, the red, lightcyan, magenta, black and midnightblue modules were negatively associated with the aforementioned traits. In order to identify the most significant genes for predicting peritoneal metastasis, the blue module was selected, which was strongly associated with peritoneal metastasis traits (Figure 2B, C). A total of 525 genes were screened from 2,633 genes according to the weight value (weight>0.3) of each gene in the blue module. GO enrichment analysis of 525 genes indicated that the extracellular matrix, proteinaceous extracellular matrix and endoplasmic reticulum lumen were the most significantly enriched pathways according to gene function annotation (Figure 2D). KEGG pathway enrichment analysis indicated that the Focal adhesion and PI3K-Akt signaling pathways were the most significant pathways. Furthermore, the TGF-beta and Wnt signaling pathways and the Proteoglycans in cancer and ECM-receptor interaction pathways were also enriched from the selected genes (Figure 2E). These results suggested that genes in the blue module were highly associated with the process of tumorigenesis.

### Identification of vital candidate marker in of peritoneal metastasis

**Figure 3:**
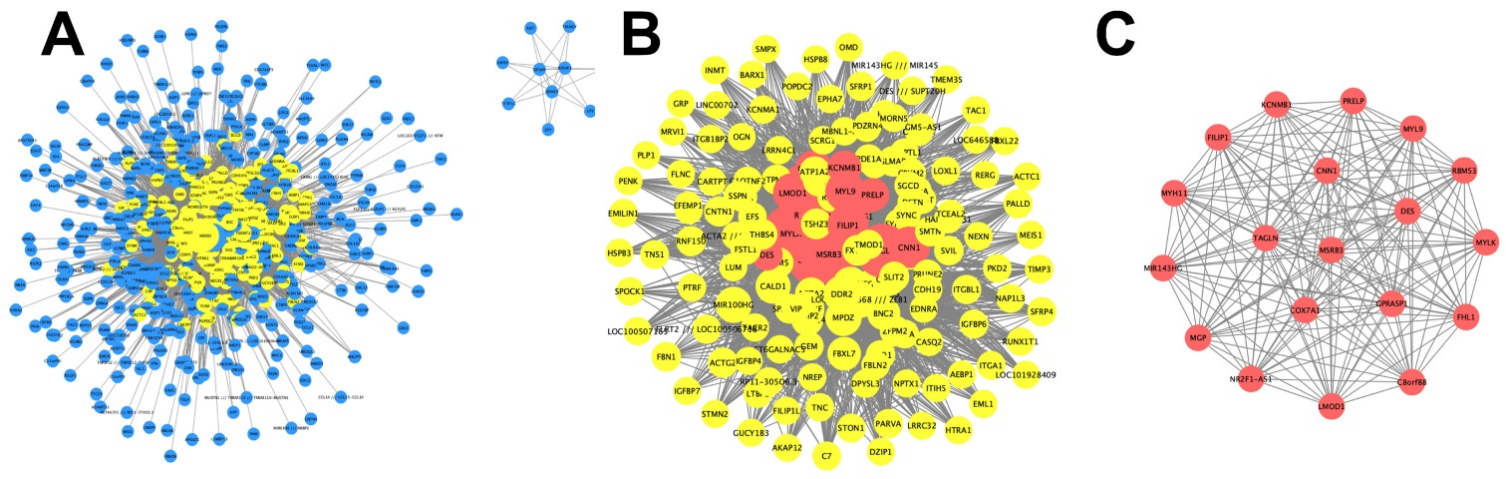
** Identification of vital candidate marker in of peritoneal metastasis.** (A) PPI network of genes in blue module and consists of 525 nodes and 6140 edges. (B) Hub network extracted from (A) by calculating topological features and consists of 167 nodes and 3633 edges. (C) Top significant sub-module cluster was identified by using MCODE algorithm.

In order to identify the vital candidate marker of peritoneal metastasis in gastric cancer, we constructed a network using PPI information, which consisted of 525 nodes and 6,140 edges (Figure 3A, Table S1). The topological features of the network were described by the three following parameters: Degree, Betweenness and Closeness. The median value of each parameter was calculated (Degree:9, Betweenness:8e-8, Closeness:0.495). The genes with Degree> 18 (2 fold of median value), Betweenness>8e-8 and Closeness>0.495 were selected to construct the hub network, which consisted of 167 nodes and 3,633 edges (Figure 3B). To further investigate the vital candidate peritoneal metastatic marker from the 167 hub genes, the dense cluster in the hub network was detected using Molecular Complex Detection. The top significant sub-module was selected from 5 clusters according to the MCODE score (Figure 3C) and the seed gene *MSRB3* was identified in the top network (Table S2).

### Prognostic value of MSRB3 in gastric cancer

**Figure 4:**
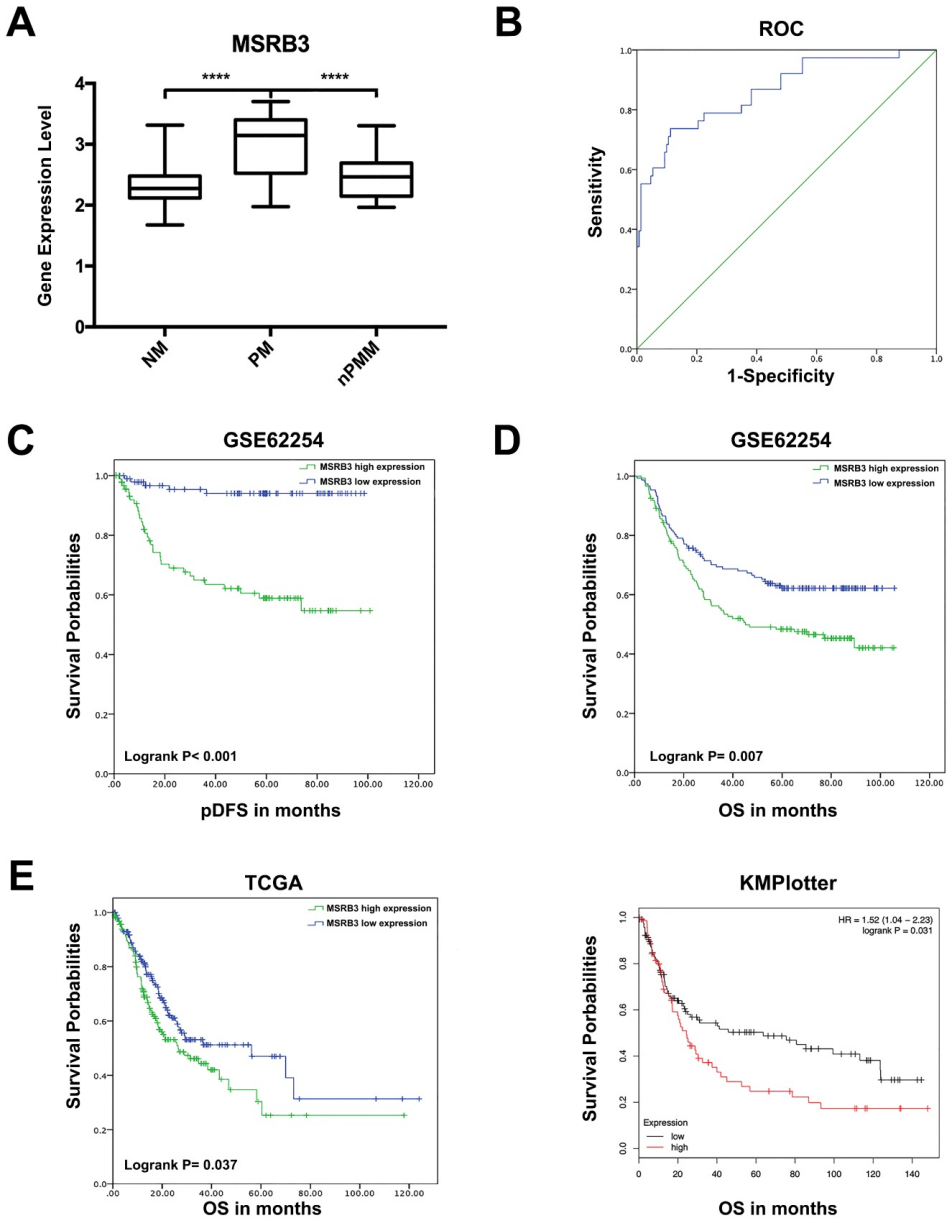
** Prognostic value of MSRB3 in gastric cancer.** (A) The expression level of MSRB3 in peritoneal metastasis group (PM), non-metastasis group and non-peritoneal metastasis group. Data are mean±SD. ****P<0.0001 (Student's t-test). (B) Receiver operating characteristic curve for MSRB3 expression level and peritoneal metastasis. (C) peritoneal Disease-Free Survival (pDFS) of MSRB3 in TCGA cohort by Kaplan-Meier (KM) analysis (log-rank P < 0.001). (D) Overall Survival (OS) of MSRB3 in TCGA cohort by Kaplan-Meier (KM) analysis (log-rank P = 0.007). (E) External validation of overal survival of TCGA and KMplotter cohort by Kaplan-Meier (KM) analysis (log-rank P = 0.037, P = 0.031, respectively).

The expression levels of *MSRB3* were significantly higher in the peritoneal metastasis group (PM) than those noted in the non-metastasis (NM, p<0.001) and non-peritoneal metastasis groups (nPM, p<0.001) (Figure 4A). To further confirm the predictive value of MSRB3 in peritoneal metastasis, 190 patients with peritoneal recurrence or without recurrence were screened form the total sample size of 295 GC patients (Table S3). The Receiver operating characteristic curve for MSRB3 expression levels and peritoneal metastasis was constructed in order to evaluate the diagnostic value of MSRB3. The area under the ROC curve (AUC) was 0.864 (95% confidence interval CI]: 0.793-0.935, p<0.001), which indicated that MSRB3 was a reliable prognostic factor for peritoneal metastasis (Figure 4B). A logistic regression model was used to analyze the risk factors for the variables peritoneal seeding and ascites positive (Table 2). MSRB3 was a significant risk factor for peritoneal metastasis in gastric cancer patients (peritoneal seed odds ratio OR]:8.467, 95%CI: 2.201-32.576; Ascites Positive OR:7.330, 95%CI: 1.853-29.002). Kaplan-Meier survival analysis of peritoneal disease-free survival (pDFS) indicated that high levels of MSRB3 were significantly associated with poor prognosis (log-rank P < 0.001) (Figure 4C). The results of univariate and multivariate analyses are shown in Table 3. MSRB3 was the independent predictor of pDFS in both univariate (HR 8.559, 95% CI; 3.339-21.937; P<.001) and multivariate analysis (HR 3.982, 95% CI; 1.509-10.509; P=.005). Furthermore, we detected the prognostic value of MSRB3 in predicting overall patient survival. Patients with high levels of MSRB3 exhibited a significantly shorter OS (log-rank P = 0.007). The external validation was performed by TCGA (log-rank P = 0.037) and KMplotter (log-rank P = 0.031) (Figure 4D, E). In summary, these results demonstrated that MSRB3 was an effective marker for predicting peritoneal metastasis and poor prognosis in gastric cancer.

### Experimental validation by gastric cancer cells

**Figure 5:**
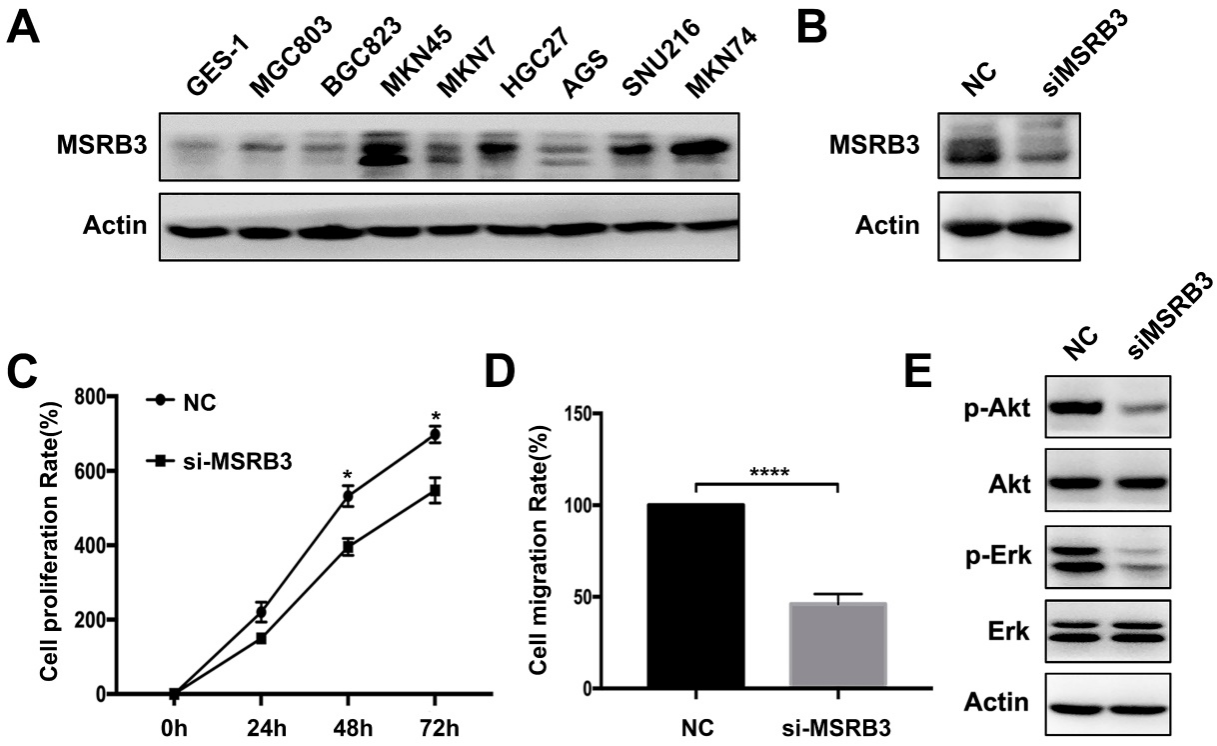
** Experimental validation by gastric cancer cells.** (A) Western blot was used to detect the expression level of MSRB3 in different gastric cancer cell lines. (B) MKN45 cell was knockdown of MSRB3 gene and western blot was used to detect the expression level of MSRB3. (C) MKN45 cell was knockdown of MSRB3 gene. MTT assay was used to detect the cell proliferation rates in 0h, 24h, 48h and 72h. Data are means ± SD in three independent experiment (*P < 0.05). (D) Transwell assay was performed to detect the migration of MKN45 cell after silencing MSRB3 for 48h. Data are means ± SD in three independent experiment (****P < 0.0001). (E) Western blot was used to assess the expression levels of phosphor-Akt, Akt, phosohor-Erk, Erk and Actin in MKN45 cells by silencing MSRB3 for 48h.

The expression levels of MSRB3 in different gastric cancer lines were tested by western blotting. The results indicated that MSRB3 was highly expressed in the MKN45 cell line compared with other cell lines. The lowest expression level was noted in the normal gastric cell line GES-1 (Figure 5A). Thus, MKN45 cell line was chosen to further analysis. In order to confirm the function of MSRB3 in the MKN45 cell line, the MTT assay was used to assess the cell proliferation rate following silencing of MSRB3 expression by small interfering RNA (siRNA) (Figure 5B). The results indicated that silencing of MSRB3 significantly suppressed tumor cell growth and restrained cell migration of MKN45 as shown in Figure 5C, D. Furthermore, depletion of MSRB3 suppressed the phosphorylation levels of Akt and Erk which were the key proteins in PI3K-AKT signaling pathway and consistent with the results of the KEGG enrichment analysis (Figure 5E). These data suggested that MSRB3 was a key protein in regulating gastric cancer cell proliferation and migration.

## Discussion

Peritoneal metastasis is one of the most common causes of death in gastric cancer. The limitation of treatment efficacy and the microinvasion of cancer cells result in poor prognosis of patients with peritoneal metastasis. Recently, several studies have focused on predicting peritoneal seeding, indicating that early detection of peritoneal metastasis is an effective way to improve clinical outcomes in gastric cancer 15-17. In the present study, we aimed to explore the predictive marker and molecular mechanism of peritoneal metastasis in gastric cancer. WGCNA is a regulatory network method based on power law distribution, which can detect gene modules and identify candidate genes associated with external parameters 18. We constructed weight networks through a soft threshold by WGCNA in the gastric cancer dataset GSE62254 derived from the NCBI datasets. As a result, 13 modules were selected and the critical module that was positively associated with peritoneal metastasis was identified. Further enrichment analysis demonstrated that genes in the blue module were involved in the Focal adhesion, PI3K-Akt, TGF-beta and Wnt signaling pathways, which are highly relevant to tumorigenesis and tumor progression. The hub genes of peritoneal metastasis were screened by measuring topological features of the PPI network, which were constructed from the genes in the blue module. Among the genes within the top significant sub-module cluster, *MSRB3* was the highest scoring gene and was marked as seed gene.

Methionine sulfoxide reductases (Msrs) are repair enzymes that reduce methionine sulfoxide residues in a stereospecific manner. They are composed of two subunits, namely MsrA and MsrB^19^. MSRB3 is one of the three MsrB enzymes in mammalian cell, acting on the R-form of methionine sulfoxide. It further plays an important role in protecting oxidative damage by eliminating cellular reactive oxygen species (ROS) 20. MSRB3 has two different forms, MSRB3A and MSRB3B, which are targeted to the endoplasmic reticulum (ER) and mitochondria, respectively 21, 22. In the present study, we confirmed that high expression levels of *MSRB3* were highly associated with peritoneal metastasis and poor prognosis in gastric cancer. Furthermore, cell validation demonstrated that *MSRB3* promoted both proliferation and migration in gastric cancer. Several studies have reported that MSRB3 deficiency can induce cancer cell apoptosis by the intrinsic mitochondrial pathway and via the modulation of the ER stress status 22, 23. In addition, MSRB3 further participates in the malignant transformation of normal mammary stem cells in breast cancer 24 and can be used as a predictor of unfavorable survival in muscle-invasive bladder urothelial carcinoma (MIBC) patients 25. However, the function of MSRB3 in cancer metastasis has not been investigated to date. The present study was the first to demonstrate that MSRB3 can participate in peritoneal invasion of gastric cancer.

The regulatory mechanism of gastric cancer metastasis by MSRB3 is unclear. KEGG enrichment analysis of the blue module indicated that the PI3K-Akt pathway was involved in mediating peritoneal metastasis. It is well known that the PI3K-Akt pathway plays a critical role in tumor proliferation and migration. Xiong et al. indicated that PRL-3 promoted peritoneal metastasis of gastric cancer by regulating PTEN via the PI3K-Akt signaling pathway 26. In addition, AKT participated in fatty-acid induced gastric cancer metastasis via the AKT/GSK-3β/β-catenin pathway 27. Fang et al. demonstrated that GC patients with PIK3CA amplifications were more likely to exhibit peritoneal metastasis compared with those without PIK3CA amplification 28. In the present study, the downregulation of the PI3K/Akt signaling pathway was confirmed in gastric cancer cells by silencing of MSRB3 expression. This suggested that the PI3K-Akt pathway participated in MSRB3-mediated peritoneal metastasis. The TGF-beta and Wnt signaling pathways were also significantly enriched. Therefore, the underlying mechanism of MSRB3 association with poor prognosis and peritoneal recurrence requires further investigation.

In summary, *MSRB3* was identified by the WGCNA approach as a critical gene in predicting peritoneal metastasis and recurrence in gastric cancer. High expression levels of *MSRB3* were noted in peritoneal metastasis patients and were associated with poor prognosis. In addition, silencing of MSRB3 expression inhibited gastric cancer cell proliferation and migration by downregulating the PI3K/Akt pathway. These results highlighted the crucial role of MSRB3 in gastric cancer and provided novel insight in the early detection of peritoneal metastasis.

## Supplementary Material

Supplementary table 1.

Supplementary table 2.

Supplementary table 3.

## Figures and Tables

**Table 1:** Characteristics of GSE62254 cohort

Characteristic	Number of Patients(%)
Age(years)	
Median(Range)	63 (24-86)
Gender	
Male	195 (66.1)
Female	100 (33.9)
T stage	
T2	184 (62.4)
T3	90 (30.5)
T4	21 (7.1)
N stage	
N0	38 (12.9)
N1	128 (43.4)
N2	79 (26.8)
N3	50 (16.9)
M stage	
M0	268 (90.8)
M1	27 (9.2)
TNMstage	
I	30 (10.2)
II	94 (31.9)
III	95 (32.2)
IV	76 (25.8)
Lauren	
Intestinal	144 (48.8)
Diffuse	134 (45.4)
Mixed	17 (5.8)
Recurrence	
Yes	124 (42)
No	153 (51.9)
unknown	18 (6.1)
First Site of Recurrence	
liver	36 (12.2)
peritonealseeding	54 (18.3)
ascitesclinicallysignificant	47 (15.9)
intraabdominal_LN	49 (16.6)
distantlymphnode	4 (1.4)
bone	7 (2.4)

**Table 2:** Univariate analysis of peritoneal metastasis in GSE62254 by logistics regression model

Characteristic	Peritoneal seed			Ascites Positive		
	P value	OR	95%CI	P value	OR	95%CI
Sex	.511	0.719	0.269-1.922	.324	1.712	0.588-4.986
Age	.020*	0.956	0921-0.993	.141	0.972	0.935-1.010
Tstage	.566	1.345	0.489-3.694	.485	1.424	0.529-3.837
Nstage	.775	0.876	0.355-2.165	.728	1.161	0.501-2.692
pTNMStage	.069	3.244	0.913-11.531	.075	3.115	0.893-10.866
Lauren	.084	2.437	0.886-6.700	.030*	3.169	1.119-8.979
MSRB3	.002*	8.467	2.201-32.576	.005*	7.33	1.853-29.002

*P<0.05; OR, odds ratio; CI, confidence interval.

**Table 3:** Univariate and multivariate analysis of peritoneal disease-free survival in GSE62254 by Cox regression model

Characteristic	No.		Univariate analysis			Multivariate analysis		
	Patients	Evens	HR	95%CI	P Value	HR	95%CI	P Value
Age(years)	190	38	0.959	0.934-0.985	0.002			
Gender								
Female	72	20	1					
Male	118	18	0.512	0.271-0.968	0.039			
T stage								
T2	122	9	1					
T3	56	23	7.56	3.495-16.357	<0.001			
T4	12	6	7.916	2.812-22.283	<0.001			
N stage								
N0	29	2	1					
N1	90	10	1.706	0.373-7.797	0.491			
N2	46	13	5.596	1.260-24.857	0.024			
N3	25	13	13.726	3.085-61.079	0.001			
TNMstage								
I	24	1	1					
II	67	3	1.051	0.109-10.120	0.966	0.453	0.045-4.521	0.5
III	61	14	6.617	0.869-50.389	0.068	1.812	0.227-14.483	0.575
IV	38	20	21.652	2.898-161.779	0.003	8.951	1.153-69.512	0.036
Lauren								
Intestinal	88	4	1					
Diffuse	94	33	8.684	3.074-24.534	<0.001	6.804	2.281-20.295	0.001
Mixed	8	1	3.753	0.419-33.605	0.237	1.719	0.189-15.627	0.631
MSRB3								
Low	98	5	1					
High	92	33	8.559	3.339-21.937	<0.001	3.982	1.509-10.509	0.005
